# Complete genome sequence of *Stutzerimonas* sp. strain CXK-1, a facultatively anaerobic bacterium isolated from an alkaline lignin-amended coastal sediment enrichment

**DOI:** 10.1128/mra.00657-26

**Published:** 2026-08-26

**Authors:** Yue Qu, Pengju Li, Xiaotong Yang, Hao Zhou

**Affiliations:** 1 Key Laboratory of Industrial Ecology and Environmental Engineering (Ministry of Education), School of Chemical Engineering, Ocean and Life Sciences, Panjin Campus, Dalian University of Technology 12399, Panjin, China; Indiana University Bloomington, Bloomington, Indiana, USA

**Keywords:** *Stutzerimonas*, alkaline lignin, complete genome sequence, aromatic compound degradation, nitrate respiration

## Abstract

We report the complete genome sequence of *Stutzerimonas* sp. strain CXK-1 isolated from coastal sediment of Liaodong Bay, China. The genome comprises a single circular chromosome of 4,133,985 bp with a G + C content of 65.03% and contains 3,810 predicted protein-coding genes.

## ANNOUNCEMENT

Lignin is an abundant aromatic biopolymer in aquatic and sedimentary environments (1, 2). Genome sequences of facultatively anaerobic lignin-enriched bacteria remain limited.

*Stutzerimonas* sp. strain CXK-1 was isolated from coastal sediment of Liaodong Bay, China (40.492°N, 122.097°E). Sediment (10 g) was inoculated into 200 mL enrichment medium in a 500-mL Erlenmeyer flask and incubated aerobically at 30°C and 150 rpm for 5 days. The medium contained NaCl (19.45 g/L), MgCl₂ (5.98 g/L), Na₂SO₄ (3.24 g/L), CaCl₂ (1.8 g/L), KCl (0.55 g/L), KBr (0.08 g/L), NaNO₃ (2.5 g/L), KH₂PO₄ (1 g/L), trace element solution (1 mL/L), and alkaline lignin (2.5 g/L) as the sole organic carbon source; agar (20 g/L) was added for solid medium. Cultures were serially diluted, repeatedly streaked, and transferred eight times. Before genome sequencing, the purified strain was cultivated aerobically for 4 days at 30°C and 150 rpm in the same liquid medium.

For preliminary identification, DNA was extracted using the Ezup Column Bacterial Genomic DNA Extraction Kit (Sangon Biotech, B518255). The 16S rRNA gene was amplified in 25-µL reactions containing 12.5 µL Taq Plus mix (BBI, B600090), 1 µL each of 10 µM primers 27F (AGTTTGATCMTGGCTCAG) and 1492R (GGTTACCTTGTTACGACTT), 1 µL DNA, and 9.5 µL ddH₂O. Cycling was 95°C for 5 min; 30 cycles of 94°C for 30 s, 57°C for 30 s, and 72°C for 90 s; and 72°C for 10 min. Amplicons were Sanger sequenced on an Applied Biosystems 3730XL. EzBioCloud identified *Stutzerimonas balearica* DSM 6083^T^ (CP007511) as the closest validly published species (99.86% similarity) (3).

Whole-genome DNA was extracted using the E.Z.N.A. Bacterial DNA Kit (Omega Bio-tek, USA) and sequenced by Shanghai Biozeron Biotechnology Co., Ltd. Illumina DNA was fragmented to approximately 450 bp using a Covaris M220, size selected, and sequenced on a NovaSeq 6000 (2 × 150 bp), generating 6,572,136 raw reads. Trimmomatic v0.36 with ILLUMINACLIP:adapters.fa:2:30:10, SLIDINGWINDOW:4:15, and MINLEN:75 yielded 6,093,396 clean reads (4). For PacBio sequencing, DNA was sheared to 8 to 10 kb using G-tubes; a SMRTbell library was prepared with the SMRTbell Express Template Prep Kit 2.0, size selected using BluePippin, and sequenced on a Sequel II using Sequencing Kit 2.0 chemistry, generating 180,104 raw reads (1,324,874,443 bp; N50, 14,858 bp).

Hybrid *de novo* assembly used Unicycler v0.5.0 with default parameters except where otherwise noted. The provider report recorded SPAdes v3.9.0 for short-read assembly, with Illumina reads used for correction and polishing (5, 6). Circlator v1.5.5 was run with default parameters to resolve the contig ends and circularize the chromosome (7); no separate manual rotation step was documented. The final genome comprised one 4,133,985-bp chromosome with a G + C content of 65.03%. NCBI PGAP revision 6.11 annotated 3,920 genes, including 3,810 protein-coding genes, 60 tRNAs, 12 complete rRNAs, 4 ncRNAs, and 34 pseudogenes. PGAP identified genes associated with benzoate/catechol conversion and the beta-ketoadipate pathway (*benABC*, *catA*, *catC*, *pcaDF*) and nitrate-associated metabolism (nitrate reductase genes, *nirBD*, and *nosZ*) (8).

## Data Availability

The complete chromosome sequence of *Stutzerimonas* sp. strain CXK-1 has been deposited in GenBank under accession number CM173217.1. The Illumina paired-end raw reads have been deposited in the Sequence Read Archive under accession number SRR38055305. The PacBio long reads have been deposited in the Sequence Read Archive under accession number SRR39795402. The BioProject and BioSample accession numbers are PRJNA1432746 and SAMN56348525, respectively. The 16S rRNA gene sequence of strain CXK-1 has been deposited in GenBank under accession number PZ819369.
